# First Report of *Clinostomum complanatum* (Trematoda: Digenea) in European Perch (*Perca fluviatilis*) from an Italian Subalpine Lake: A Risk for Public Health?

**DOI:** 10.3390/ijerph17041389

**Published:** 2020-02-21

**Authors:** Vasco Menconi, Chiara Manfrin, Paolo Pastorino, Davide Mugetti, Luana Cortinovis, Elisabetta Pizzul, Alberto Pallavicini, Marino Prearo

**Affiliations:** 1 The Veterinary Medical Research Institute for Piemonte, Liguria and Valle d’Aosta, Via Bologna 148, 10154 Torino, Italy; vasco.menconi@izsto.it (V.M.); davide.mugetti@izsto.it (D.M.); marino.prearo@izsto.it (M.P.); 2 Department of Life Sciences, University of Trieste, via Giorgieri 10, 34127 Trieste, Italy; cmanfrin@units.it (C.M.); pizzul@units.it (E.P.); pallavic@units.it (A.P.); 3 DVM, 24021 Albino (Bergamo), Italy; lucortinovis89@gmail.com

**Keywords:** clinostomidae, epidemiological survey, public health, zoonoses

## Abstract

*Clinostomum complanatum*, a digenean trematode of the Clinostomidae family, is a fish-borne zoonotic parasite responsible for Halzoun syndrome in humans and is transmitted through the consumption of raw or undercooked freshwater fish. Of the total of 112 specimens of European perch (*Perca fluviatilis*) sampled from a subalpine lake (Lake Endine) in North Italy in 2019, 21 (18.75%) tested positive for encysted metacercariae in the fillet. This study reports the first isolation of *C. complanatum* in *P. fluviatilis* and highlights the possible zoonotic risk for consumers, since *P. fluviatilis* is a food fish used in the traditional local cuisine.

## 1. Introduction

*Clinostomum complanatum* (Rudolphi, 1814) is a digenean trematode of the Clinostomidae family. The high degree of morphological differences within the genus *Clinostomum* and similarities among species have resulted in several taxonomic revisions of *Clinostomum* species [1]. Trematodes of the genus *Clinostomum* are endoparasites with a complex life cycle involving two intermediate hosts and one definitive host [2]. The life cycle of *C. complanatum* is similar to other species in the Clinostomidae family [3]: the adult stage resides in the buccal cavity of piscivorous birds and reptiles but rarely mammals, including humans. The first intermediate hosts are freshwater gastropods and the second are fish and amphibian species [4]. In the first intermediate host, miracidia hatch from eggs and undergo asexual reproduction several times before developing into sporocysts, rediae, and then brevifurcate cercariae. Free-swimming cercariae penetrate the skin of fish, where they develop into metacercariae that are infectious for piscivorous birds, the definitive hosts [5].

*C. complanatum* is a fish-borne zoonotic parasite responsible for Halzoun syndrome, a rare disease transmitted after consumption of raw or undercooked freshwater fish infected with metacercariae [6,7,8,9]. In accidental human infections, *C. complanatum* attaches to the mucous membrane of the throat and generally causes acute pharyngitis and laryngitis [10]. Tiewchaloern et al. [11] reported an unusual case of eye infection caused by *Clinostomum* sp. Following the initial human infection by *C. complanatum* described by Yamashita [6], other cases have been described in countries where consumption of raw fish is part of the traditional cuisine, although laryngitis caused by *C. complanatum* is a rare disease [10]. Since there are no drugs for the treatment of clinostomiasis, surgical extraction should be promptly performed under general anesthesia [12].

Subalpine lakes are important ecosystems for recreational tourism and for water supplies in agriculture and fisheries [13]. The European perch (*Perca fluviatilis*) is an invaluable resource for local fishers. It is largely used in preparing raw and cooked dishes by local people and restaurants. With this study, we report the first isolation of *C. complanatum* in *P. fluviatilis* from Lake Endine, a subalpine lake in Lombardy, northern Italy.

## 2. Materials and Methods 

### 2.1. Study Area

**Figure 1:**
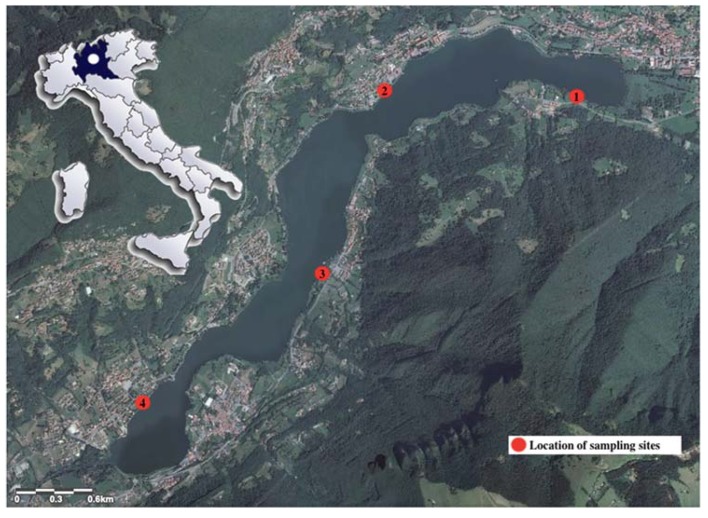
Lake Endine (Lombardy, northern Italy) and location of sampling sites (in red): site 1 (45°47′15.3″ N 9°57′53.3″ E); site 2 (45°47′22.1″ N 9°56′58.0″ E); site 3 (45°46′25.2″ N 9°56′26.5″ E); site 4 (45°45′46.2″ N 9°55′21.5″ E).

Lake Endine is a subalpine lake (surface area 2.3 km^2^, perimeter 14 km, maximum depth 9.4 m) located at 337 m a.s.l. in Lombardy (Province of Bergamo, northern Italy) (Figure 1). Fish assemblage is typical of the lentic environment and includes *Scardinius erythrophthalmus*, *Perca fluviatilis*, *Carassius carassius*, *Cyprinus carpio*, *Tinca tinca*, *Squalius cephalus*, *Esox lucius*, and *Silurus glanis*.

### 2.2. Fish Sampling, Anatomopathological and Morphological Examination 

European perch were captured by local fishers using fishing rods from four sites in the littoral zone of Lake Endine in 2019 (from spring to summer) (Figure 1). The sampling was part of a monitoring campaign conducted by the Fish Diseases Laboratory of the Istituto Zooprofilattico Sperimentale del Piemonte, Liguria and Valle d’Aosta (IZSPLV, Turin, Italy) to check the sanitary condition of wild fish within a research project funded by the Italian Ministry of Health.

The fish were put in cold boxes at 4 °C and transferred to the IZSPLV laboratory for analysis. The fish were weighed (g), measured for total length (cm), sexed, and subjected to anatomopathological and parasitological examination for the presence of zoonotic parasites. Body surface, fins, abdominal cavity, gills, eyes, and skeletal muscle were carefully examined. For parasitological examination of the fillet, each flesh side was divided into four sections: anteroventral (belly flap), anterodorsal, posteroventral, and posterodorsal. Internal organs were removed and placed in Petri dishes containing saline solution. Encysted metacercariae were carefully retrieved and transferred to saline solution and excysted by breaking the cyst wall with a fine needle. Isolated parasites were rinsed in deionized water and counted. Preliminary morphological identification of the metacercariae was performed by light microscopy (Olympus BX40, Olympus, Tokyo, Japan) and fixed in 70% ethanol for molecular analysis. Specimens for morphological examination were clarified in Amman’s lactophenol solution following the protocol described by Caffara et al. [14].

### 2.3. Molecular Analysis

The genomic DNA was extracted from a single specimen using E.Z.N.A.® Insect DNA kit (Omega, Bio-Tek, Norcross, GA, USA) by following the manufacturer’s instructions and quantified with a Nanodrop 2000 (Thermo Scientific, Austin, TX, USA). To perform species identification, 18S primers targeting the V4 region were used (TAReuk454FWD1 and TAReukREV3) (Macrogen, Seoul, Korea) [15].

PCR reactions were performed on samples using the AccuStart II PCR SuperMix (Quanta bio, Beverly, MA, USA), and following the touch-down thermal cycle: initial denaturation 94 °C for 3′; 10 cycles with denaturation at 94 °C for 20″, annealing at 57 °C for 30′ and elongation at 72 °C for 1′, then 25 cycles with denaturation at 94 °C for 20″, annealing at 47 °C for 30″ and elongation at 72 °C for 1′. Each PCR reaction was performed in a final volume of 20 μL, with a final concentration of 1 X supermix, 0.4 μM of each primer and 1 μL of DNA (average DNA concentration 22 ng/µL). 

PCRs were sent to an external service to be Sanger sequenced (Eurofins, Hamburg, Germany). The consensus sequence obtained has been deposited at GenBank under accession number MN982880.

### 2.4. Statistical Analysis

Prevalence, mean intensity and mean abundance of the infestation were calculated according to Bush et al. [16]. Differences in the prevalence of infestation for the four sampling sites were tested for using the Chi-Square Test. Spearman’s rank correlation coefficient (ρS) was used to test for correlations between biometric characteristics (total length and total weight) and sex and presence of metacercariae. The criterion for significance was set at *p* < 0.05. Statistical analyses were performed using open source data analysis software RStudio® version 1.1.463 (RStudio, Inc., Boston, MA, USA).

## 3. Results

A total of 112 specimens (28 from each sampling site) of *P. fluviatilis* were caught during 2019 (Table 1) and examined for the presence of encysted metacercariae. No visible lesions in external and internal organs were observed.

A total of 21 fish (18.75%) tested positive for the presence of metacercariae in the fillet. The prevalence of infestation in the four sampling sites ranged from 14.3% (site 1) to 21.4% (sites 3 and 4), the mean intensity from 1 (site 1) to 2 (site 4) metacercariae, and the mean abundance from 0.14 (site 1) to 0.42 (site 4) metacercariae (Table 2). A total of 28 metacercariae were isolated from muscle tissue: 12 from the anterodorsal, 2 from the posterodorsal, 3 from the anteroventral, and 12 from the posteroventral section. No difference in the prevalence of infestation for the four sampling sites was noted (*p* = 0.8861).

Spearman’s rank correlation did not show any correlations between biometric characteristics and sex and the presence of metacercariae.

Metacercariae were clearly visible to the naked eye and varied in color from pale yellow to yellow; all were morphologically identified as *C. complanatum* [2,17,18,19].

The PCR sequencing of the V4-18S region for species identification confirmed the identity of *C. complanatum* by BlastN on Digenea taxid:6179 with *C. complanatum* sequences (GenBank IDs FJ609420 andAY245701) all supported by an e-value: 0 and an identity of 99%. Before using the Stoeck et al. primers [15], PCR was initially run using the classical Folmer primers [20], obtaining an amplicon with a low identity of 90.7% with *Schistosoma mansoni* (e-value 3e11).

## 4. Discussion

This is the first study to report the presence of *C. complanatum* in fillets of *Perca fluviatilis* from a subalpine lake in Italy. Samplings of fish fauna (mainly *P. fluviatilis*) in other Italian subalpine lakes (Iseo, Como, Garda, and Maggiore) for the presence of *Diphyllobothrium latum* have detected no other zoonotic or potential zoonotic agents to date [21]. The present study shows that *Perca fluviatilis* is a suitable intermediate host for *C. complanatum*. Because the parasite is described as a zoonotic agent, knowing its prevalence and distribution range is of particular importance for the local economy and for consumer health [6,8,9,10,22].

Molecular analysis confirmed the identity of *C. complanatum* by using the primers from Stoeck et al. [15] but those from Folmer et al. [20] failed to correctly identify the species.

European perch is widely distributed throughout the Northern Hemisphere [23]; it is host for a wide range of endoparasitic helminths [24] and zoonotic agents [21,25,26]. The species holds commercial interest as well as economic importance for recreational fishing in many European lake systems including lakes in Italy [13]. European perch is used in the local cuisine of northern Italy, where it is generally consumed marinated raw as *carpaccio di pesce persico*. This may result in adverse consequences for consumer health.

Human clinostomiasis has been reported in America, Southeast Asia, and eastern Europe [27], but not in Italy. *C. complanatum* was found in wild fish from freshwater streams in northern and central Italy [18]. Among these were fish of interest for conservation and not of interest for commercial value, such as barbel (*Barbus* spp.) and chub (*Squalius cephalus*).

The metacercariae of *C. complanatum* are known to cause considerable damage to the viscera and the musculature of fish species [2,28,29]. However, we found a low mean intensity of infestation (1.38), and anatomopathological examination disclosed no lesions or alterations in internal organs. The mean intensity of infestation in *P. fluviatilis* reported by Çolak et al. [25] from Lake Sığırcı (Edirne, Turkey) and by Soylu et al. [30] from Lake Gala (Edirne, Turkey) (2.8 and 2.4, respectively) are higher compared to our data. Again, we found no lesions or alterations in internal organs or muscle tissue. Massive infection in fish generally causes weight loss, atrophy due to the mechanical effects of the metacercariae on the surrounding tissue, fish mortality, as well as unmarketability of the infected specimens [2,31,32]. These phenomena should also be considered for biodiversity conservation and commercial fishing sustainability since *P. fluviatilis* is an important food source for the local economy.

The prevalence of infection was similar to that reported by Çolak et al. [25] (Edirne, Turkey) and Kadlec et al. [33] from the Morava river basin, Czech Republic (13.1% and 15%, respectively) but much lower than the prevalence reported by Soylu et al. [30] (53.8%) for Gala Lake (Turkey). Gala Lake was declared a national park in 2005; it lies on the main avian migratory route and provides a breeding area for native and migrant birds, including fish-eating birds [30,34]. This may contribute to the high prevalence recorded for Gala Lake.

Scientific opinion on the risk assessment of parasites in fishery products [35] has emphasized the need to define the risk for consumers based on epidemiological studies investigating fish-borne zoonoses. Clinostomiasis is not listed in the document, however, because epidemiological studies performed before its publication were lacking.

Lake Endine has several conditions that make it favorable for the maintenance of the *C. complanatum* life cycle: it is shallow in depth (max. 9.4 m) and it hosts several mollusks, fish, and bird species involved in the parasite’s life cycle. Furthermore, the shallow water with high concentrations of gastropods and fish attracts swarms of piscivorous birds simultaneously, thus perpetuating the parasite’s cycle [5]. The ecological factors involved in its life cycle are not entirely known, however. Furthermore, the possible role of *Perca fluviatilis* as a source of human infection in Italy will need to be further investigated in other lakes and watercourses in order to obtain new epidemiological data that can be used to inform prevention and control measures by public health authorities.

In response to the increasing popularity related to the consumption of marinated, raw, or partly cooked fish dishes, health authorities have implemented measures to reduce food-borne illness [35,36,37]. However, these measures remain targeted only for certain zoonotic infections. New tools, technologies, and public awareness initiatives including health education figure among the options for the effective monitoring, control, and prevention of food-borne parasitic zoonoses.

## 5. Conclusions

Parasites are widespread in all ecosystems. Among vertebrates, fish have the highest rates of parasitic infection owing to their aquatic environment [38]. In response to the increasing popularity of raw fish dishes, researchers and public authorities are called to implement measures to prevent food-borne parasitic diseases. Effective regulations, as well as systems and tools for the routine diagnosis and monitoring of zoonotic parasites, are fundamental public health activities that can reduce the risk of disease transmission to consumers. While much attention has focused on monitoring the large subalpine lakes (Garda, Como, Iseo, and Maggiore), the present study highlights the need to monitor small lakes as well, since they may provide a reservoir for zoonotic parasites. Further studies are needed to create a risk map for zoonotic parasites in Italian lakes. While the promotion of food safety and hygiene measures that can be taken—either by food establishments or by consumers themselves—are available for zoonotic infections such as anisakiasis or diphyllobothriasis, no data are available regarding the effectiveness of this in *C. complanatum* metacercariae. Thus, further studies and regulations are needed in the near future.

## Figures and Tables

**Table 1:** Sampling site, total length (mean ± SD), total weight (mean ± SD), and sex (male—M or female—F) of fish (N) sampled in 2019.

Sampling Site	N	Total Length (cm)	Total Weight	Sex (%)	
			(g)	M	F
1	28	14.15 ± 3.44	43.73 ± 27.78	25	75
2	28	15.27 ± 4.34	45.78 ± 22.47	30	70
3	28	14.46 ± 2.53	43.80 ± 26.98	27	73
4	28	13.12 ± 3.24	41.63 ± 27.78	28	72

**Table 2:** Sampling site, prevalence, mean intensity, and mean abundance of infestation.

Sampling Site	Prevalence (%)	Mean Intensity	Mean Abundance
1	14.3	1	0.14
2	17.8	1.2	0.21
3	21.4	1.16	0.25
4	21.4	2	0.42
